# Effect of Sex, Body Mass Index and Physical Activity Level on Peak Oxygen Uptake Among 14–19 Years Old Adolescents

**DOI:** 10.3389/fspor.2020.00078

**Published:** 2020-06-30

**Authors:** Boye Welde, Bente Morseth, Bjørn Helge Handegård, Pål Lagestad

**Affiliations:** ^1^Faculty of Health Sciences, School of Sport Sciences, UiT The Arctic University of Norway, Tromsø, Norway; ^2^RKBU Nord, Faculty of Health Sciences, UiT The Arctic University of Norway, Tromsø, Norway; ^3^Department of Physical Education and Sport Science, Faculty of Teacher Education and Art, Nord University, Levanger, Norway

**Keywords:** body mass index, cardiorespiratory fitness, exercise, longitudinal changes, physical activity, VO_2peak_, youth

## Abstract

The aim was to describe longitudinal trends in peak oxygen uptake (VO_2peak_) among 14- to 19-year-old adolescents in Norway, and to examine effects of sex, body mass index (BMI), and physical activity (PA) level on VO_2peak_ during adolescence. Of 124 invited students from two lower secondary schools in Norway, 116 eighth-grade students (61 boys and 55 girls; 14 years old at baseline) volunteered to participate. The study has a longitudinal design with 6 yearly repeated measures of body height and mass, VO_2peak_ and PA level. VO_2peak_ allometrically scaled to body mass raised to the power of 0.67 was measured using a walking or running incremental test on a treadmill, whereas PA level was self-reported. Among 696 possible observations, 555 (79.7%) were valid. Multiple linear regression and linear mixed model analyses were used to examine the associations between age, sex, BMI, PA level and VO_2peak_. VO_2peak_ showed a non-linear pattern from age 14 to 19, with a distinct increase for boys peaking at age 17, while the results provide a flatter and more stable curve for girls. Sex, BMI and PA level together explained 43–71% of the variance in VO_2peak_ at the different age levels. Sex and PA level contributed independently to explain a significant proportion of the variance in VO_2peak_ at all measurement occasions, while BMI did not. Adjusted sex differences in VO_2peak_ increased over time, from 26.5 ml·kg^−0.67^·min^−1^ at age 14 to 55.5 ml·kg^−0.67^·min^−1^ at age 19. The independent contribution from PA level to the variance in VO_2peak_ increased from age 14 to 16 and then decreased. While PA level explained 32.5% of the total variance in VO_2peak_ for 16-year-olds, this number was 14% in 19-year-olds. In conclusion, aerobic power showed a non-linear pattern during adolescence, peaking at age 17. Sex and PA level explained a large proportion of the variance in VO_2peak_, each of them being an independent contributor to VO_2peak_. Aerobic power is linked to improved health and seems to depend largely on sex and PA level in adolescents, emphasizing the importance of maintaining a sufficient PA level during adolescence.

## Introduction

Cardiorespiratory physical fitness (CRF), frequently quantified as maximal oxygen uptake (VO_2max_) (Bassett and Howley, 2000), has shown to be strongly and inversely related to risk of cardiovascular disease, diabetes, and mortality in both adolescents and adults (Ortega et al., 2008; Carnethon et al., 2009; Kokkinos and Myers, 2010; Dencker et al., 2012; Buchan et al., 2015; DeFina et al., 2015; Myers et al., 2015; Bangsbo et al., 2016). Moreover, low CRF is a predictor of premature all-cause and cardiovascular mortality independently of other risk factors, similar to that for cigarette smoking and elevated cholesterol levels (Kokkinos and Myers, 2010). However, there is also reported weak associations between CRF and health outcomes, suggesting that the associations between CRF normalized for body mass and health are largely explained by fat mass (Ried-Larsen et al., 2013; Haapala et al., 2020). Thus, it is suggested that the gold standard method of defining CRF is to scale it by lean body mass using ratio standards or allometric procedures (Loftin et al., 2016).

However, adolescence is an important age period for habit forming into adulthood (Howie et al., 2016), and detailed knowledge of CRF and its association to age, sex, and physical activity level during this period is essential. A systematic review found that there has been a substantial decline in the CRF among adolescents during the last decades (Tomkinson et al., 2019). Preventive efforts aimed at maintaining physical fitness and physical activity level through puberty are shown to have favorable health benefits in later years (Janz et al., 2000; Aarnio et al., 2002; Telama et al., 2006; Loprinzi et al., 2012).

Studies measuring CRF in youth and adolescents show an influence of age and sex. In existing studies from Norway and Denmark, VO_2max_ levels vary from 52 to 59 ml·kg^−1^·min^−1^ in boys and 40 to 49 ml·kg^−1^·min^−1^ in girls (Andersen et al., 1987; Kolle et al., 2010; Nes et al., 2013). The differences in VO_2max_/VO_2peak_ observed in these studies may partly be due to different test protocols. In a study of Andersen et al. (1987), the boys had ≈18% higher VO_2max_ values than the girls per kg lean body mass, which lead the authors to suggest that girls of this age have the lower fitness level. There is agreement that VO_2peak_ values are higher in boys than girls (McMurray et al., 2002, 2003; Armstrong et al., 2011; Kemper et al., 2013). Research have shown an increase in VO_2peak_ in absolute terms (L·min^−1^, ml·min^−1^) among adolescents up to around 14 years of age for girls and 17 years of age for boys (McMurray et al., 2002; Armstrong et al., 2011; Kemper et al., 2013). Armstrong et al. (2011) report that boys almost doubled their absolute VO_2peak_ from 11 to 17 years, while girls increased about 50% in the same age range.

However, VO_2peak_ relative to body mass (ml·kg^−1^·min^−1^) declines as the youth ages (McMurray et al., 2002, 2003; Armstrong et al., 2011; Kemper et al., 2013). This is least pronounced among boys, whereas girls exhibit a steady decrease in VO_2peak_ values when expressed relative to body mass (Armstrong et al., 2011). This may be due to that fat mass naturally increases in girls during pubertal development, leading to a relatively decline in muscle mass, whilst in boys the lean muscle mass increases and hence facilitates both the utilization of oxygen by the muscles and indirectly augments the stroke volume of the heart through increased venous return (Armstrong et al., 2011). In late adolescence, VO_2max_ per kg lean body mass showed no reduction from age 16 to 19 years in boys, while the girls demonstrated a small reduction over this age span (Andersen et al., 1987).

Moreover, CRF seems to be closely linked to overweight and obesity, as well as physical activity level (Åstrand et al., 2003; Ornelas et al., 2011; Marta et al., 2012; Nes et al., 2013; Porter et al., 2017; Gonsalves et al., 2018). In boys and girls followed from 9 to 17 years of age, changes in CRF predicted changes in body fat percentage (Ornelas et al., 2011). In boys, CRF improved over time while body fat percentage decreased, whereas CRF decreased and body fat percentage increased among girls. CRF explained 39% of the total variance in waist circumference, indicating a strong relationship (Ornelas et al., 2011. Furthermore, CRF is generally positively associated with physical activity level, both self-reported and objectively measured (Kemper and Koppes, 2004; Emaus et al., 2010; Marta et al., 2012; Nes et al., 2013; Dyrstad et al., 2016; Porter et al., 2017). However, in adults, a recent study showed that large variations in objectively measured physical activity level, were reflected in small variations in VO_2max_, and the correlation between physical activity level and VO_2max_ was moderate (*r* < 0.50) (Dyrstad et al., 2016). In adolescents, studies show small to moderate correlations between VO_2max_ and activity level (*r* = 0.30 – 0.34) (Michaud et al., 2002; Lubans et al., 2008). However, most studies have estimated VO_2max_ from submaximal tests and applied pedometers as measure of physical activity level (Michaud et al., 2002; Lubans et al., 2008; Ottevaere et al., 2011).

Most longitudinal studies of physical activity level during adolescence show a decline from around 13 years of age (Anderssen et al., 2005; Kjønniksen et al., 2008; Reilly, 2016; Steene-Johannesen et al., 2019), some as large as 30 percent points decrease from age 13 to age 19 years (Anderssen et al., 2005). In other studies, a large decline in physical activity level has been found between 8 and 16 years of age (Kimm et al., 2000; Telama and Yang, 2000; McMurray et al., 2003). However, one study in middle-school girls reported stable physical activity levels between the age 12 and 14 years (Baggett et al., 2008). Taking the positive correlation between physical activity level and CRF into consideration (Alves et al., 2016, 2017), the decline in adolescents' physical activity level is worrying. Interestingly, Kemper and Koppes showed a strong cross-sectional association between physical activity and VO_2max_, but physical activity level was not a strong predictor of future change in CRF in a longitudinal study, following adolescents from the age of 13 to the age of 36 (Kemper and Koppes, 2004).

Present research emphasize the importance of examining the effect of sex, body mass index (BMI) and activity level, in order to understand the variance in longitudinal development of VO_2peak_ levels among adolescents. The aim of this study was therefore to examine longitudinal trends in VO_2peak_, properly scaled to body mass, among 14- to 19-year-old adolescents in Norway, and to explore how sex, BMI and physical activity level affect VO_2peak_ during adolescence. It was hypothesized that sex and physical activity level affects VO_2peak_ at all ages. More specifically, we hypothesized that: (1) increased physical activity level affects VO_2peak_ positively; (2) boys will have a higher VO_2peak_ when controlled for body mass than girl at all ages; (3) physical activity level decreases from the age of 14 to the age of 19.

## Materials and Methods

### Design

Data from an observational study which included participants from six randomly selected classes from two lower secondary schools in Norway were used to examine the research question. The study included longitudinal measures of VO_2peak_ and questionnaire data of self-reported physical activity level.

### Subjects

**Figure 1:**
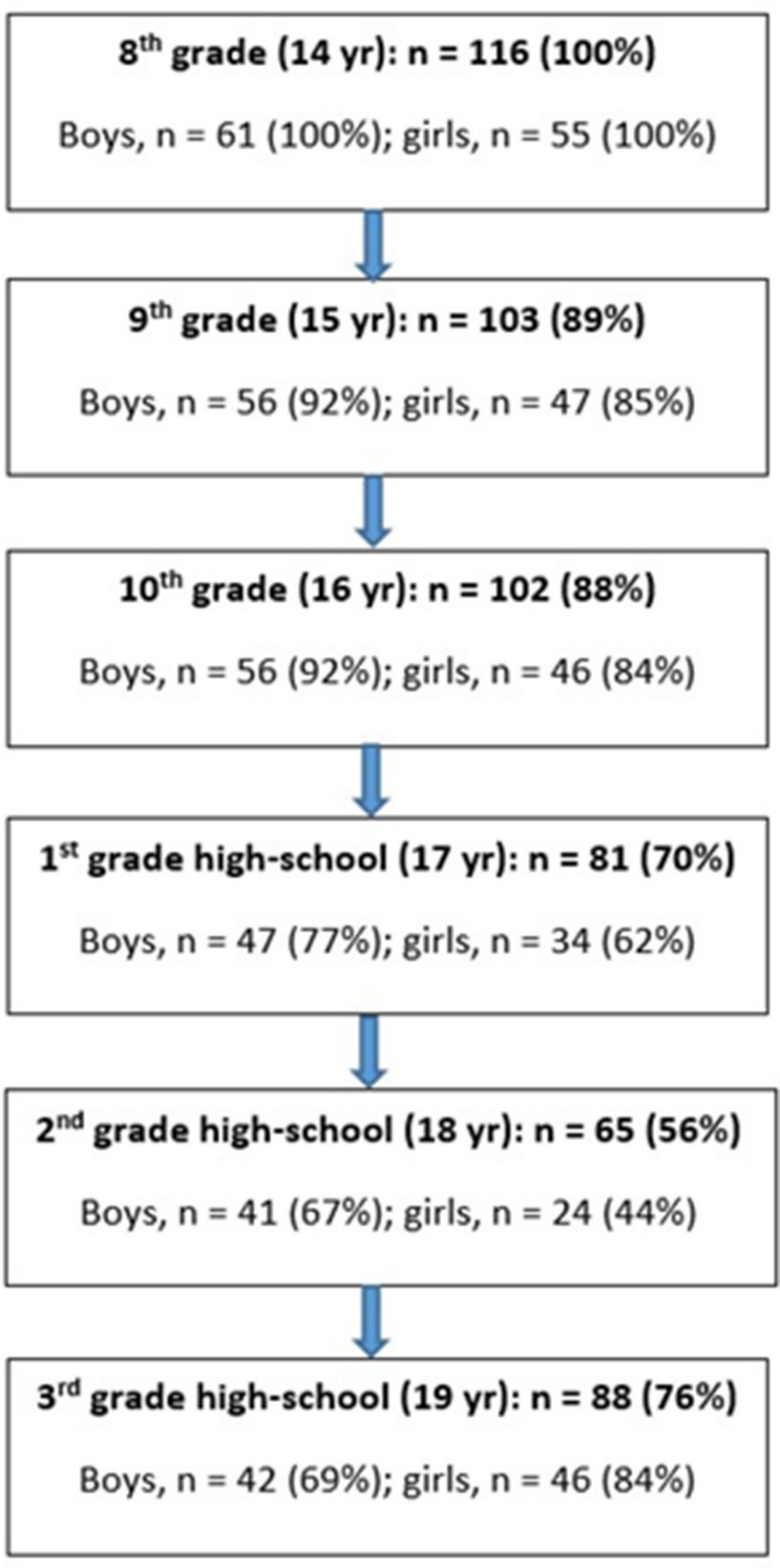
Flow-chart showing the number of subjects tested each year.

The participants were recruited from six randomly selected classes from two lower secondary schools in a town in the middle of Norway. Of the 124 students, 116 eighth-grade students volunteered to participate in this study (age = 14.0 ± 0.5 yr, body mass = 54.2 ± 10.9 kg, height = 1.63 ± 0.08 m). The distribution of boys and girls was relatively equal in the sample (61 boys and 55 girls), as well as the distribution of urban and rural students. Figure 1 shows the number of students with valid test data at each year during the data collection period. Students dropped out of the study for various reasons—illness, injury, pregnancy, and relocation—thereby explaining the different number of subjects tested each year. A subject could re-enter the study after dropping out 1 year. Out of a total of 696 possible observations (116 subjects tested six times), 555 observations (79.7%) were valid, and 141 observations (20.3%) were missing. There were 303 valid observations for the boys (83%) and 252 valid observations for the girls (76%). The subjects were fully informed about the study and its protocols before giving their informed written consent to participate in this study in accordance with the Declaration of Helsinki, and were made aware that they could withdraw from the study at any point without providing an explanation. Approval to use the data and conduct the study was given by the Norwegian Center for Research Data and the Norwegian Regional Committees for Medical and Health Research Ethics (REK 488715, 2014).

### Procedures

Questionnaire responses of self-reported physical activity and measurements of physical fitness (VO_2peak_), height, and body mass were obtained from each subject at the same time in each examination. Data collections occurred during a 2-month period (April–May) each year when the students were in the 8th grade (14 years old), 9th grade (15 years old), 10th grade (16 years old), and also at first year (17 years old), second year (18 years old) and third year (19 years old) at high school. Location, test leaders, equipment, and procedures were similar throughout the study.

Height was measured with a stadiometer (Kawe, NorEngros, Oslo, Norway) that was permanently connected to the wall. The subjects did not wear shoes, and height was converted to the nearest centimeter. Body mass was measured using Seca Digital Weight Scales (Seca Gmbh & Co., Germany, Model 877, accuracy of 0.1 kilo). Body mass index was calculated by dividing body mass (kg) by height (cm) squared, in accordance with international standards (Cole et al., 2000).

Oxygen uptake measurements were carried out as walking or running on a treadmill (Woodway S5; Woodway, Waukesha, WI, USA). The number of individuals in the test lab at any time was limited to the test leader and one student, in order to keep the oxygen level in the air stable and to avoid disturbances during the tests. The test and measurement procedures were repeated verbally to the subjects before each test. The subjects were advised to avoid strenuous exercise the day before testing, and to eat 2–3 h before testing. The students were also informed that they could participate in school physical education (PE) before testing, but that only light activity was permitted (≤75% HR_max_, maximal duration of 45 min). The test outfit consisted of running shoes, shorts or training pants, and a T-shirt or jumper.

Oxygen uptake and other respiratory variables were measured continuously with the Oxycon Pro (Erich Jaeger GmbH, Hoechberg, Germany), set to operate in mixing chamber mode. The metabolic system was carefully calibrated, including a two-point gas calibration (ambient air and a mixture of 16.00% O_2_ and 5.00% CO_2_), and calibration of the inspiratory flow meter using the automatic system. The ambient room air was checked routinely before and after each test. The treadmill had an incline of 10.5% during tests to prevent the subjects' running technique from being a limiting factor for the maximum oxygen uptake. Before arriving at the laboratory, the students carried out a detailed 15 min warm-up procedure by themselves. When at the laboratory, the students were asked how much they trained. Girls who did not train or were obese started with a speed of 4 km/h, those who trained one to two times a week, started with a speed of 5 km/h, while those who trained more than two times a week started with a speed of 6 km/h. The same categories were used for boys, but at a 1 km/h higher speed. The treadmill speed was increased by 1 km/h every minute (or 0.5 km/h at the final stage of the test) until volitional exhaustion. The criteria for a test to be considered maximal, was a leveling-off in oxygen uptake (increased <2 mlO_2_·kg^−1^·min^−1^), and a respiratory exchange ratio ≥ 1.05. The leveling-off criteria was seen in 95% of the tests, while in 73% of the tests the RER-criteria was achieved. Hence, since not all of the students demonstrated one or both of these criteria, the term VO_2peak_ was used in this study. The average of the two highest 30-s consecutive measurements determined VO_2peak_. The test had a duration time of 5–6 min. In children and adolescents it is important to adjust CRF for differences in body mass using scaling procedures, as it may not be reasonable to assume that VO_2peak_ is proportional to body mass using the power of 1, specifically during the years of pubertal development. Hence, in this study, VO_2peak_ is expressed in accordance to Åstrand et al. (2003), who states that the theoretically derived value is 0.67 based on dimensional theory. This scaling factor is also close to what Nes et al. (2013) found by allometric regression in a study sample of boys and girls, a sample similar to our subjects regarding age (13–18 years), and being from the same municipal as our subjects.

At the end of the test protocol, the students answered a questionnaire used among adolescents, where a question about physical activity level was also included (Rangul et al., 2008): “How many days a week are you physically active that you become sweaty or out of breath?” The response options were “never,” “1 day a week,” “2–3 days a week,” “4–5 days a week,” and “6–7 days a week.” The question about physical activity level was recoded into a new variable with the options; 0, 1, 2.5, 4.5, and 6.5, to represent number of days with physical activity.

### Statistical Analysis

Multiple regression was used to assess the relationship between VO_2peak_ and sex, BMI, and the number of active days per week, in separate analyses for each measurement occasion.

We used linear mixed model analysis (LMM; Multilevel analysis; Singer et al., 2003) to examine longitudinal effects of self-reported physical activity level on VO_2peak_. In LMM we utilize the fact that data is hierarchical, where repeated observations (level 1) are nested within individuals (level 2). Physical activity level is a predictor measured repeatedly over time, and LMM is suited to handle time-varying predictors.

In LMM, all observations are retained in the analysis through the use of full information maximum likelihood estimation (FIML). To evaluate statistical significance, a significance level of 0.05 was used. Model fit for two nested models was evaluated by comparing −2log likelihood (LL) for each model, using the chi-square distribution with degrees of freedom equal to the difference in parameters estimated in the two models. For example, we tested whether adding the square of (age-−14) to the level 1 part of the model would improve fit by the same procedure as suggested by Singer et al. (2003). Since the trajectory of the VO_2peak_ curve seems to follow a curve that is non-linear in time, we made this comparison.

Statistical analyses were performed with SPSS statistical software version 24 (IBM SPSS, Chicago, IL, USA).

## Results

Since it may not be reasonable to assume that VO_2peak_ in adolescents is proportional to body mass raised to the power of 1, we explored the scaling exponents in our sample separate for each sex, by using allometric regression. The regression estimates (unstandardized coefficients, ß) were significantly different from zero, and 0.67 was included in 11 of the 12 95% CI's for ß. These values, given the 95% CI's for both sexes and all measurement occasions, approximate the theoretically derived value of 0.67 (Åstrand et al., 2003), and, hence, a common exponent was applied to both sexes; each individual's absolute VO_2peak_ (mL·min^−1^) was divided by body mass raised to the power of 0.67. This scaled expression of VO_2peak_ was not significantly correlated with body mass within the various age groups, except for girls at the age of 14 (*r* = −0.27, *p* < 0.05). This indicates that appropriate adjustment for body mass was performed in our analysis, however, with a tendency that the coefficient for boys (0.79–0.88) may be higher than for girls (0.38–0.60).

### The Effect of Sex, BMI, and Activity Level on Allometrically Scaled VO_2peak_

Table 1 shows the descriptive data for the variables included in the study, separately for each sex on each measurement occasion. We tested, separately for each measurement occasion, the strength of the association between VO_2peak_ and sex, BMI, and the number of active days per week using multiple regression. As a group, these three predictors explained from 43 to 71% of the variance in VO_2peak_ at the different age levels (Table 2). Sex and activity level contributed independently to explain a significant proportion of variance in VO_2peak_ at all measurement occasions, while BMI did not when body mass was scaled to the power 0.67. Controlling for BMI and activity level, the adjusted difference in VO_2peak_ between boys and girls were between 26.5 ml·kg^−0.67^·min^−1^ at age 14, and 55.5 ml·kg^−0.67^·min^−1^ at age 19. After taking sex and BMI into account, increasing exercise days by 1 day gives an estimated increase in VO_2peak_ between 4.4 and 9.3 ml·kg^−0.67^·min^−1^, with the lowest effect for 14 and 19-year-olds.

**Table 1:** Means (and standard deviations) for allometrically scaled VO_2peak_, number of active days per week, and BMI for each sex and measurement occasion.

**Age**	**14**		**15**		**16**		**17**		**18**		**19**	
**Variable**	**Boys (*n* = 61)**	**Girls (*n* = 55)**	**Boys (*n* = 56)**	**Girls (*n* = 47)**	**Boys (*n* = 56)**	**Girls (*n* = 46)**	**Boys (*n* = 47)**	**Girls (*n* = 34)**	**Boys (*n* = 41)**	**Girls (*n* = 24)**	**Boys (*n* = 42)**	**Girls (*n* = 46)**
VO${}_{2\text{peak}}^{\text{a}}$	193.6 (24.0)	161.2 (22.7)	212.2 (24.8)	167.6 (26.2)	215.5 (28.4)	163.1 (24.2)	229.3 (30.4)	171.0 (28.6)	213.1 (29.4)	157.5 (24.0)	216.4 (26.8)	156.5 (18.8)
BMI^b^	19.3 (2.8)	21.1 (3.8)	20.2 (3.2)	21.6 (3.6)	20.8 (3.5)	22.2 (3.7)	22.0 (4.0)	23.3 (4.5)	22.6 (4.0)	23.9 (4.5)	23.0 (4.1)	23.2 (4.1)
Activity level^c^	3.7 (1.8)	2.9 (1.6)	3.2 (1.7)	2.8 (1.8)	3.2 (2.2)	2.6 (1.6)	3.5 (2.0)	3.1 (2.0)	3.2 (1.8)	2.9 (1.6)	3.7 (1.9)	3.0 (1.7)

a *Measured in milliliters of oxygen per (kilogram of body mass)^−0.67^ per minute*.

b Height/weight^2^ measured in kg/m^2^.

c *Coded as number of days with increased heart rate or sweating per week*.

**Table 2:** Multiple regression with sex, activity level, and BMI predicting allometrically scaled VO${}_{2\text{peak}}^{\text{a}}$.

**Age**	**14**	**15**	**16**	**17**	**18**	**19**
**Predictor**	**(*n* = 105)**	**(*n* = 97)**	**(*n* = 101)**	**(*n* = 81)**	**(*n* = 65)**	**(*n* = 86)**
Sex	26.47^**^	38.76^**^	44.38^**^	54.09^**^	51.73^**^	55.50^**^
BMI	−1.17	−0.70	−0.76	−1.24	−0.92	−0.52
Activity level	4.38^**^	6.91^**^	8.54^**^	8.81^**^	9.34^**^	5.14^**^
R^2^^b^	0.43	0.58	0.71	0.67	0.66	0.68

* p < 0.01,

** p < 0.001.

Note. The table contains unstandardized regression coefficients.

a Measured in milliliters of oxygen per (kilogram of body mass)^−0.67^ per minute.

b *Proportion of the total variance in allometrically scaled VO_2peak_ explained collectively by sex, activity level, and BMI*.

### Longitudinal Development of Allometrically Scaled VO_2peak_ in the 14–19 Age Range

**Figure 2:**
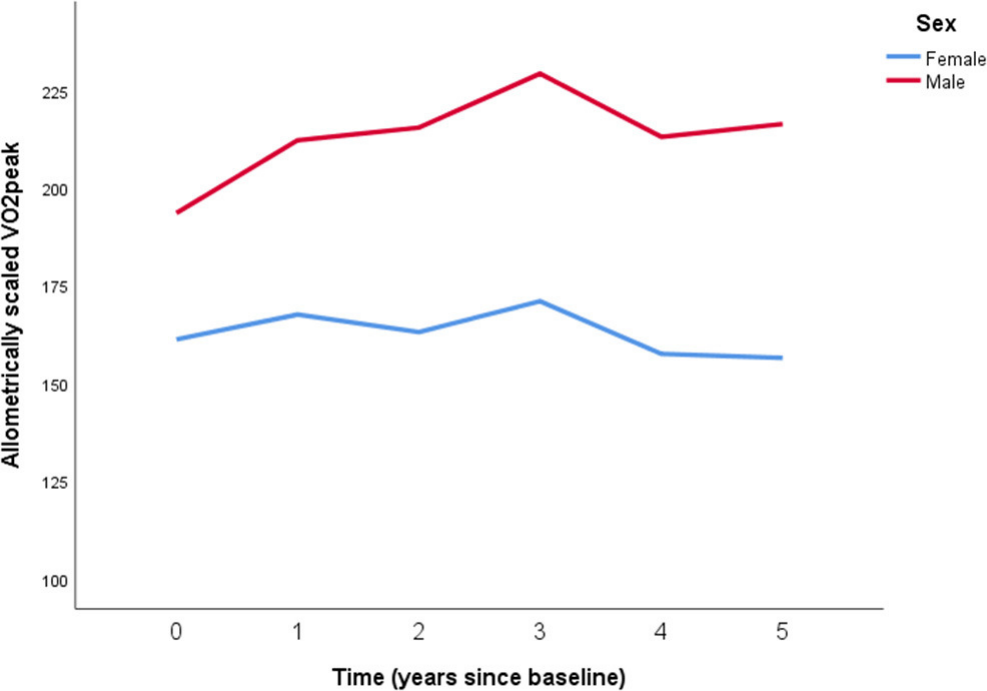
Longitudinal development of peak oxygen uptake (VO_2peak_) with body mass allomterically scaled to the power of 0.67 for 61 boys (red line) and 55 girls (blue line) from the age of 14–19. Overall mean and pooled SD for boys: 212.4 ± 29.1 mlO_2_·kg^−0.67^·min^−1^. Overall mean and pooled SD for girls: 162.8 ± 24.3 mlO_2_·kg^−0.67^·min^−1^. Time is years from baseline: Time 0 = 8th grade, Time 1 = 9th grade, Time 2 = 10th grade, Time 3 = 1st year high school, Time 4 = 2nd year high school, Time 5 = 3rd year high school.

Figure 2 indicates a non-linear pattern in the development of aerobic power over the age span. In addition, when inspecting individual line diagrams, the most common trajectory showed a slight increase in VO_2peak_ over the first 3 or 4 years, and then a slight decrease. This pattern was most evident for boys.

To assess whether a non-linear model (with time and time squared as level 1 predictors) has better model fit than a linear model (with only time as a level 1 predictor), we compared the model fit for these two models. When adding the square of time to the model, the −2·LL changed from 4917.89 to 4766.07, and this change was highly significant. This indicates improved model fit of the non-linear model (χ^2^(4) = 150.82; *p* < 0.0001), suggesting that the rate of change over the 14–19 age range is not constant, and that this non-linearity should be incorporated into other models assessing longitudinal effects on VO_2peak_. The non-linear model had significant residual variation on level 1, indicating a potential for more level 1 predictors, such as activity level, to be included in the model. In addition, when inspecting random effects there was significant variation in the individual trajectories, opening up for the inclusion of level 2 predictors such as sex.

### Sex Effects on Longitudinal Allometrically Scaled VO_2peak_ Scores

Table 2 indicates that the regression coefficients for sex increase with increasing age. We tested whether we have sufficient evidence to claim that sex differences in VO_2peak_ increase with time using a linear mixed model analysis. We compared two models: (1) A full model including time·sex and time squared·sex interactions (−2LL = 4448.90), and (2) A reduced model without these two interaction terms (−2LL = 4491.84). In both models BMI and activity level were included as time-varying covariates.

The difference in −2LL, 4491.84–4448.90 = 42.94 (df = 2), is significant (*p* < 0.001), so we have evidence to say that sex differences varies over time, and that separate tests show that sex differences in allometrically scaled VO_2peak_ increase with time.

### Activity Level as a Predictor of Longitudinal Allometrically Scaled VO_2peak_ Scores

We wanted to test whether activity level affects VO_2peak_ differently over 6 years of adolescence, and for potential differences between boys and girls regarding this association. Since VO_2peak_ changes non-linearly over the age segment in this study, we wanted to assess whether activity level affects the curvature of the longitudinal trajectory differently over time. Body mass index was included as a time-varying control variable in these tests, even though it was not significantly associated with the allometrically scaled VO_2peak_ in the separate test on each measurement occasion.

Therefore, we fitted a linear mixed model, where number of active days per week (activity level), BMI, Time, Time squared, and the interaction between time and number of active days per week, and the interaction between time squared and number of active days per week, were added as level 1 predictors. On level 2, Sex was included as a predictor for all level 1 parameters except for the parameter associated with BMI. Residuals for the intercept, and for the slope and curvature parameters were included in the model.

To test sex differences in how the strength of association between number of active days per week and allometrically scaled VO_2peak_ varies over time, we compared two models: (1) A full model including two three-way interaction terms (time by activity level by sex; time squared by activity level by sex), and (2) a reduced model without those three-way interactions. When adding the two three-way interactions to the model, the −2*LL* changed from 4436.29 to 4435.93, and this change was non-significant [$\chi_{\left(2\right)}^{2}=0.36;p>0.05].\;$Therefore, there is no evidence of sex differences in how the activity level affects the change rate of VO_2peak_.

We then tested whether the association between VO_2peak_ and activity level varied over time (for both sexes combined) by comparing two models: (1) A model that included interaction terms between the time variables and activity level (time by activity level; time squared by activity level), and (2) A model without those interaction terms. Adding the two interaction terms involving time and activity level to the model, the −2*LL* changed from 4448.69 to 4436.29, and this change was significant (χ^2^(2) = 12.40;*p* = 0.002). This significant result gives us reason to state that there is evidence that the strength of association between VO_2peak_ and activity level varies over time. Inspecting the correlation between VO_2peak_ and activity level for different time points, give some clues of how to interpret this. The strength of association increases from age 14 to 16 and then decreases. While activity level explains 32.5% of the total variance in allometrically scaled VO_2peak_ for 16-year-olds, this number is 14.9% for 19-year-olds.

## Discussion

The main finding in this study was the presence of a slight increase in relative VO_2peak_ at the age of 14 to the age of 17 among boys, whereas for the girls the relative VO_2peak_ was stable from the age of 14 to the age of 17. From age 17 to 19 years, VO_2peak_ decreased in both sexes. Moreover, sex differences in VO_2peak_ increased with time, and the strength of association between VO_2peak_ and activity level varied over time, with an increase from the age of 14 to the age of 16, and thereafter a decrease. Furthermore, sex and activity level, but not BMI, were highly significant predictors of VO_2peak_, as both sex and activity level independently explained a significant proportion of variance in VO_2peak_ on all measurement occasions.

Although relative VO_2peak_ is commonly normalized to body size (ml·kg^−1^·min^−1^), the choice of body size measure is debated (Lolli et al., 2017). VO_2peak_ can be scaled to whole body mass (ml·kg^−1^·min^−1^) or an allometric exponent, for example 2/3 (ml·kg^−0.67^·min^−1^), or relative to fat-free mass (Lolli et al., 2017). As VO_2peak_ relative to body mass may not adequately account for differences in body size when investigating longitudinal changes in VO_2peak_ among adolescents, and direct measures of lean body mass were not available in our study, we used an allometric exponent (ml·kg^−0.67^·min^−1^) in an attempt to optimize the expression of VO_2peak_ in relation to body mass (Pettersen et al., 2001). The theoretically derived value is 0.67 based on dimensional theory (Åstrand et al., 2003) and this scaling factor is close to what Nes et al. (2013) found by allometric regression in a study sample of boys and girls who was quite similar to our subjects regarding the age span (13–18 years).

It could also be questioned whether the VO_2peak_ tests in our study were truly maximal and if the criteria used to define a maximal effort is appropriate. Our protocol for testing VO_2peak_ included a 15 min warm-up followed by the 5–6 min incremental exercise test. The vast majority of tests (~95%) fulfilled the criteria of a leveling-off in oxygen uptake and in 73% of the tests the achieved criteria for RER ≥ 1.05 was achieved. Since the subjects performed until exhaustion and had a lower oxygen uptake than could be estimated due to running velocity and inclination at the time of terminating the VO_2peak_ test, we believe that the effort has been maximal and not submaximal.

It has been argued that the use of secondary criteria (e.g., RER) to verify a maximal effort in young people during a ramp protocol may result in the acceptance of a submaximal VO_2max_ (Barker et al., 2011). However, Barker et al. (2011) tested 9–10 year old children during cycling, while our 14–19 year old subjects ran on a treadmill. Treadmill running utilizes a larger muscle mass than cycling, which relies heavily on the work performed by the knee extensors, and by running to exhaustion it is likely that the cardiovascular, pulmonary, and metabolic systems has been stressed to a large extent in our subjects and, thus, reflects a maximal effort. Also, already in (Åstrand, 1952) showed that children often reach the criteria of RER ≥ 1.05 and, therefore, we suppose that the reliance of RER as a secondary criteria is reasonably reliable in 14–19 year old adolescents. Additionally, in our data there is no evidence toward that higher RER-values are associated with higher VO_2peak_-values and, further, there were no significant associations between the development of VO_2peak_ over follow-up between those achieving the RER-criteria and those who did not. However, in this study, we used VO_2peak_ as measure because not all participants reached commonly used criteria for VO_2max_ (i.e., RER ≥ 1.05, leveling-off in oxygen uptake).

Our findings showed a slight increase in allometrically scaled VO2_peak_ from the age of 14 to 17 years among boys. Thereafter, their VO_2peak_ fell markedly from the age of 17 to 18, and then plateaued. Among the girls, VO_2peak_ was relatively stable from the age of 14 to the age of 17. Thereafter, they mirrored the pattern seen among boys. These findings are consistent with the 2011-review by Armstrong et al. (2011), when body mass is appropriately controlled for. The slight increase in VO_2peak_ among boys during the age of 14–17 is somewhat in contradiction to other longitudinal studies (McMurray et al., 2002; Kemper et al., 2013). Kemper et al. (2013) found that VO_2peak_ relative to body mass decreased over the age range 12–17 in both sexes, but did not include adolescents at the age of 18 and 19 years of age. However, the absolute VO_2peak_ in the study of Kemper et al. (2013) increased over time in the same manner for both sexes as seen in our study. McMurray et al. (2002) found that VO_2peak_ relative to body mass was relatively stable from the age of 8 to the age of 16, but that absolute VO_2peak_ increased in both sexes. However, in their study, VO_2peak_ was predicted from a three-stage cycle ergometer test, and the study did not include VO_2peak_ measures at the age of 17, 18, and 19 years of age.

Our study showed that VO_2peak_ differed between boys and girls, and that sex differences in relative VO_2peak_ increased with time. The difference in VO_2peak_ level between boys and girls in our study is in accordance with previous studies (McMurray et al., 2002; Armstrong et al., 2011; Kemper et al., 2013; Nes et al., 2013). Girls' absolute VO_2peak_ (l·min^−1^) has been shown to be about 10% lower than those of boys during childhood, and the sex difference reached approximately 35% by age 16 years (European College of Sport Science, 2018). However, the present study presents new knowledge of longitudinal sex differences in VO_2peak_ (ml·kg^−0.67^·min^−1^) during the age period of 14–19 years.

In accordance with other studies (Richards et al., 2009; Aires et al., 2011; Marta et al., 2012; Porter et al., 2017), this study showed that adolescents' physical activity level affects CRF (VO_2peak_) positively. Moreover, in our study there was no evidence of sex differences in how the activity level affects the change of VO_2peak_. This is in line with a study by Richards et al. (2009) but in contrast to Porter et al. (2017) who found a more evident association between physical activity and CRF in girls than in boys. However, we found that the strength of association between VO_2peak_ and activity level varied over time, and to our knowledge, no other studies have examined this issue. Our findings indicate that while activity level explained one third of the total variance in VO_2peak_ for 16-year-olds, this number was much lower (15%) for 19-year-olds. Poor CRF during adolescence has been found to be associated with persistent inactivity during adolescence (Richards et al., 2009), and the results points toward the importance of a high activity level during early years of adolescence.

## Strengths and Limitations of the Study

The major strength of the study is the longitudinal design with six repeated examinations, employing the same questions and tests every year, performed using similar procedures, test equipment and test leaders. Furthermore, VO_2peak_ and BMI measurements are based on directly measured, high-quality standard procedures. However, despite that our scaling of VO_2peak_ by body mass raised to the theoretical exponent of 0.67 for both sexes largely removed the association between body mass and fitness, we have a relatively small sample and our estimates of the exponent must be interpreted with caution. Nevertheless, our use of the exponent of 0.67 is in line with Nes et al. (2013) who investigated 570 boys and girls in the same age group and from the same municipal as our sample. It should be mentioned here that we have not powered the study with the aim of accurately estimating this exponent. However, this was not our purpose for the study either. Moreover, information on physical activity level is based upon self-reported data, which is shown to overestimate real activity levels (Emaus et al., 2010), and may weakens the association between VO_2peak_ and physical activity level. Use of objective measurements instruments such as accelerometers has the advantage of decreasing subjectivity and eliminating bias such as social desirability and recall problems. Admittedly, we did not determine pubertal status of our subjects, even though we are aware that maturation and controlling for its effect on the results is of importance in longitudinal studies. We calculated retrospectively maturation offset according to Moore et al. (2015), but since the results overall remained the same as with our original variable for time and that the prediction uncertainty for individual predictions may be quite large, we chose not to include maturation offset in our analyses.

## Conclusion

The present study examined the effect of sex and physical activity level on VO_2peak_, and longitudinal development of VO_2peak_ among 14–19 years old adolescents in Norway. The rate of change in VO_2peak_ over the 14–19 years age range showed a slight increase in VO_2peak_ over the first 3 or 4 years and then a slight decrease, especially among boys. Sex and physical activity level each explained VO_2peak_ independently, and collectively these predictors explained 43–71% of the variance in VO_2peak_. The activity level explained one third of the total variance in VO_2peak_ for 16-year-olds, while this number was 15% for 19-year-olds. Finally, the direction of association between VO_2peak_ and activity level varied over time. The study confirms the importance of sex and activity level as highly significant predictors of CRF among adolescents. The findings point to the importance of maintaining a high physical activity level among adolescents. Further research should include interventions aimed at maintaining a sufficient physical activity level during adolescence, in order to maintain VO_2peak_ at a higher level.

## Data Availability Statement

The datasets generated for this study are available on request to the corresponding author.

## Ethics Statement

The studies involving human participants were reviewed and approved by the Norwegian Centre for Research Data and the Norwegian Regional Committees for Medical and Health Research Ethics (REK 488715, 2014). Written informed consent to participate in this study was provided by the participants' legal guardian/next of kin.

## Author's Note

Preliminary results from this study were presented at the 23rd annual Congress of the European College of Sport Science in Dublin, Ireland, 04.07.2018. Conference paper: European College of Sport Science (2018).

## Author Contributions

All authors listed have made a substantial, direct and intellectual contribution to the work. BH conducted the statistical analyses. BW drafted the manuscript. All authors critically revised the manuscript, gave final approval, and agree to be accountable for all aspects of work ensuring integrity and accuracy.

## Conflict of Interest

The authors declare that the research was conducted in the absence of any commercial or financial relationships that could be construed as a potential conflict of interest.
